# Biosynthesis of Copper Nanoparticles From Seaweed Ulva lactuca and Their In Vitro Antioxidative Potential

**DOI:** 10.7759/cureus.48985

**Published:** 2023-11-18

**Authors:** Shanmugam S B, Sivaperumal Pitchiah, Vasugi Suresh, Pasiyappazham Ramasamy

**Affiliations:** 1 Department of Physiology, Saveetha Dental College & Hospitals, Saveetha Institute of Medical and Technical Sciences, Saveetha University, Chennai, IND; 2 Department of Prosthodontics, Saveetha Dental College & Hospitals, Saveetha Institute of Medical and Technical Sciences, Saveetha University, Chennai, IND; 3 Department of Research, Polymer Research Lab, Centre for Marine Research and Conservation, Saveetha Dental College & Hospitals, Saveetha Institute of Medical and Technical Sciences, Saveetha University, Chennai, IND

**Keywords:** ulva lactuca, uv spectroscopy, antioxidant, innovative, cu nanoparticles, biosynthesis

## Abstract

Background: Marine macroalgae is consumed by individuals in several regions, including Scandinavia, Great Britain, Ireland, China, and Japan; in Japan, it is commonly referred to as aosa. Copper nanoparticles are primarily composed of copper and exhibit a size distribution ranging from 1 to 100 nm. Copper nanoparticles can be synthesized using chemical or natural means, similar to other nanoparticle variants. The nanoparticles in question have garnered significant attention owing to their historical utilization as coloring agents, as well as their contemporary applicability in medicine and antibacterial treatments.

Objectives: The objective of this study was to investigate the biosynthesis of copper nanoparticles derived from *Ulva* *lactuca *seaweed and explore their in vitro antioxidative potential.

Materials and methods: Seaweed samples (10 g) were mixed with 50 ml of distilled water and placed in a shaker for two days. Copper sulfate (10 mM) was mixed with 100 ml of distilled water to obtain a copper (Cu) solution. Cu nanoparticles were then synthesized by adding the aqueous extract to 100 ml of the Cu solution and mixing it in an orbital shaker at 180 rpm for 24 hours. They were observed both visually and via ultraviolet (UV) spectrophotometry to confirm their nanoparticle synthesis. The initial reading was performed using a UV-visible spectrometer at 300-800 nm. The sample was centrifuged at approximately 8000 rpm for 15 minutes, the pellet was removed, and the pellet was dried in a hot-air oven. The synthesized Cu nanoparticles were then investigated using in vitro antioxidant assays.

Result: The seaweed-derived copper nanoparticles exhibited a 1.2 peak absorbance at 580 nm. Various concentrations of copper nanoparticles (25, 50, 75, and 100 µg/ml) were tested for free radical scavenging. As the copper nanoparticle concentration increased, the scavenging ability on 1, 1-diphenyl-2-picrylhydrazyl (DPPH) radicals assay showed that the free radical scavenging activity increased in a dose-dependent manner. Similar to the DPPH assay, the total antioxidant and hydrogen peroxide (H_2_O_2_)assays showed increased free radical scavenging with increasing concentration.

Conclusion: The application of Cu nanoparticles in the synthesis process has the potential to enhance the antioxidant activity of *Ulva* *lactuca *as evidenced by the observed increase in antioxidant capacity and defense against reactive oxygen species.

## Introduction

The genus *Ulva*, which encompasses edible green algae species commonly referred to as seaweeds, exhibits a wide distribution throughout the coastal regions of several oceans worldwide. *Ulva lactuca*, derived from the Latin term for lettuce, serves as the representative species for the genus *Ulva*. Seaweed, also known as sea lettuce, can grow either in a detached, free-floating manner or in association with rocks and shells, usually through a small disc-shaped structure called a holdfast. In some instances, seaweeds can proliferate in large quantities. In the field, there is potential for confusion between *Ulva* and *Ulvaria*, which are two distinct genera of green, sheet-like seaweeds [1]. Owing to its extensive distribution and comparable prevalence, the latter, in particular, can be erroneously identified as seaweeds. *Ulva* exhibits a greater thickness than *Monostroma* and *Ulvaria,* as the latter two species are characterized by a single-cell layer thickness. The fingerprint test can be employed as a means of identification in cases of uncertainty. By examining the visibility of one's fingerprints through a translucent frond, it is possible to discern between *Monostroma* and *Ulvaria* species. If the frond lacks visible fingerprints and exhibits a texture reminiscent of that of wax paper, it is most likely to be *Ulva*. The taxonomic classification of the species previously assigned to the genus *Enteromorpha* has been revised, and it is now classified under a different genus [2].

A variety of aquatic organisms, such as manatees and sea hares (sometimes referred to as sea slugs), consume seaweeds as part of their diet. Seaweed is enjoyed by individuals in several regions, including Scandinavia, Great Britain, Ireland, China, and Japan, and is referred to as aosa in Japan. Seaweed is consumed by humans in both raw form, as an ingredient in salads, and cooked form, as an ingredient in soups. It is rich in protein, soluble dietary fiber, and several vitamins and minerals, such as iron [3]. The maximum size of *Ulva's* individual blades reaches 400 mm, albeit primarily observed when the plants are situated in sheltered environments. The organism in question is a macroscopic alga that exhibits a range of green hues that are anchored in position by a disc-shaped holdfast. Their structure is a flattened, leaf-like thallus. The thallus is a foliose structure, measuring up to 30 cm (12 inches) in length, and consists of two layers of cells. Its morphology resembled that of lettuce leaves. The object was surrounded by a rigid gelatinous casing. The holdfast structure, which serves to attach the algae to its substrate, exhibits a disc-like morphology. The life cycle involves the alternation of similar generations that undergo spore production (diploid) and gamete production (haploid). Accidental fragmentation is commonly employed as a mechanism for asexual reproduction [4].

Large amounts of hazardous hydrogen sulfide gas were created by the decomposing leaves. In one occurrence near Saint-Michel-en-Grève, Brittany, France, a horse rider passed out while hauling a load of decomposing seaweeds, crashed, and died; in another, a lorry driver was driving a load of seaweeds when he passed out, crashed, and died, with toxic gases being the alleged cause [5]. Environmentalists attribute this phenomenon to an excessive amount of nitrogenous compounds that are dumped into the ocean because of poor pig and poultry animal waste disposal on industrial farms. Various types of seaweeds are frequently encountered in the saltwater aquarium trade, where the plants are prized for their superior nutritional absorption and palatability. Many reef aquarium keepers grow seaweed species as food for herbivorous fish or utilize them in refugia. Seaweed can survive under a variety of lighting and temperature settings and is relatively simple to maintain. Seaweeds can be floated in water or fastened to live rock or other surfaces in the refugium [6].

Cu nanoparticles range in size from 1 to 100 nm and are composed of Cu. Cu nanoparticles can be synthesized chemically or naturally, similar to many other types of nanoparticles [7,8]. These nanoparticles have a long history of use as coloring agents and in biological and antibacterial applications, making them particularly intriguing. Unique properties of copper nanoparticles, including catalytic and antifungal/antibacterial actions, are not observed in commercial copper. First, copper nanoparticles exhibit extremely potent catalytic activity because of their substantial catalytic surface area [9,10]. When used as reagents in organic and organometallic syntheses, nanoparticles have a greater reaction yield and a quicker reaction time because of their small size and high porosity. While commercial Cu only showed 43 % conversion to biphenyl, Cu nanoparticles utilized in the condensation reaction of iodobenzene reached approximately 88 % conversion [9,11].

Cu nanoparticles are microscopic in size, possess a notable surface-to-volume ratio, and can function as agents with antifungal and antibacterial properties. Antibacterial activity is caused by the tight interaction between bacterial membranes and the metal ions released into the solution [12,13]. The gradual oxidation of nanoparticles in liquids can lead to the release of cupric ions, which in the presence of a surrounding lipid barrier might generate hazardous hydroxyl free radicals [14,15]. Subsequently, membrane degeneration is initiated by the oxidation of lipids within cell membranes by free radicals. Consequently, disruption of cellular membranes leads to the release of internal cellular components, thereby impairing their ability to sustain fundamental metabolic processes. Cell death occurs as a consequence of the internal alterations induced by free radicals [16,17]. Hence, the present investigation aimed to synthesize Cu nanoparticles from the marine seaweed *Ulva lactuca* and investigate their in vitro antioxidative potential.

## Materials and methods

Preparation of aqueous extract

**Figure 1:**
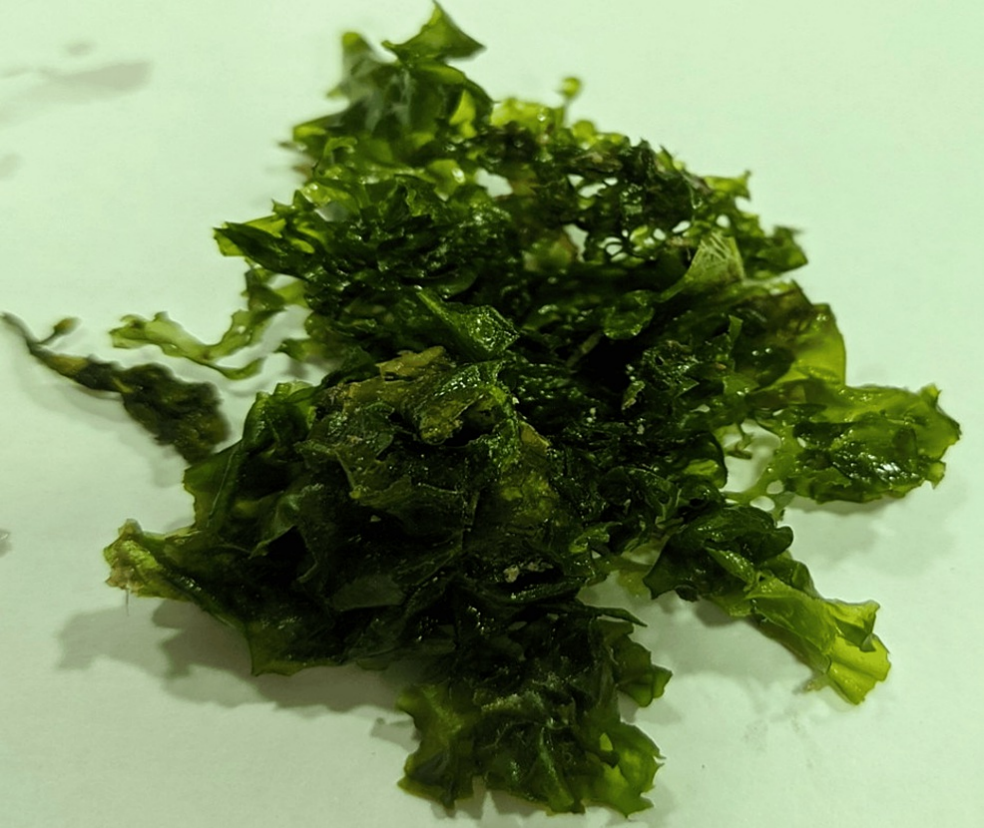
Marine seaweed Ulva lactuca

**Figure 2:**
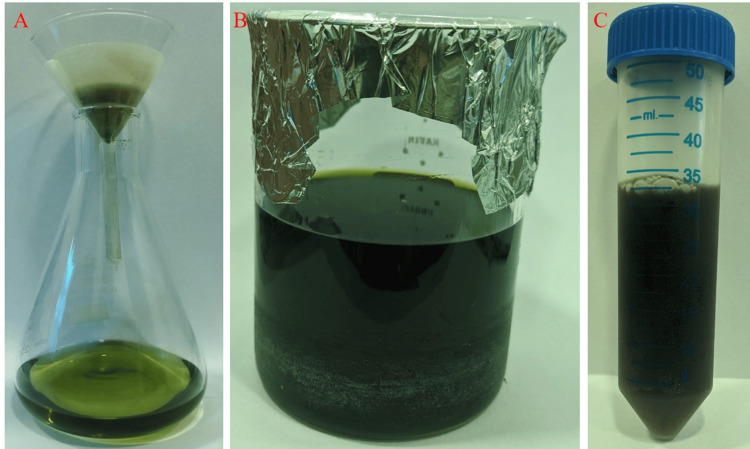
Filtration (2A), aqueous extract of marine seaweed Ulva lactuca (2B), crude extract (2C)

The study was done at the Department of Physiology, Saveetha Dental College and Hospitals, Saveetha Institute of Medical and Technical Sciences (SIMATS), Saveetha University, Chennai, India. The seaweed sample, identified as *Ulva lactuca* (Figure 1), was obtained from the Tuticorin area of Tamil Nadu, India. The pre-processing stage involved the use of distilled water for cleaning and washing the sample. The preprocessed sample was subjected to a drying procedure in a hot air oven at a temperature set below 60 ℃. After drying, the sample was pulverized into a rough powder using a mortar and pestle. Then, 50 g of the seaweed sample was introduced into a conical flask containing 100 ml of distilled water. The mixture was then agitated for approximately 24 hours (Figure 2), after which the resulting extract was passed through a muslin cloth for filtration [18].

Synthesis of copper nanoparticles

An aqueous solution of Cu sulfate (CuSO_4_) (10 mM) was prepared using double distilled water. A volume of 100 ml of CuSO_4_ solution was introduced into a conical flask, and afterward, 5-10 ml of aqueous extract was added dropwise with continuous stirring using an orbital shaker at 180 rpm for 24 hours. The biosynthesized solution was subjected to visual inspection and further evaluation using a 580 nm UV spectrophotometer. The biosynthetic samples were then centrifuged at 12,000 rpm. The pellet was maintained in isolation and subjected to a drying process using a hot air oven [19].

In vitro antioxidant activity

Scavenging Ability on DPPH Radicals

The antioxidant capacity of alkaloids was assessed by measuring their ability to scavenge the stable DPPH free radical following the methodology described by Ramasamy et al. [20]. In this experiment, different concentrations of the alkaloid (ranging from 25 to 100 µL) were combined with 2900 µL of methanol solution containing 120 µM DPPH. The mixture was then incubated in the absence of light at a temperature of 37°C for a duration of 30 min. The absorbance was measured at a wavelength of 517 nm. The percentage inhibition (I%) of free radicals by DPPH was calculated using the equation given below.

Percentage of Inhibition (I %) = (A blank - A sample) / A blank ×100

Here A blank is the absorbance of the control reaction, and A sample is the absorbance of the test compound. BHT and ascorbic acid were used as positive controls, and all tests were performed in triplicate.

Total Antioxidant Activity (TAA)

The overall antioxidant activity of the alkaloids derived from the marine actinobacterial strain was measured using the procedure outlined by Ramasamy et al. [20] with slight modification. Briefly, a sample volume of 0.3 ml was generated at various concentrations ranging from 25 to 100 µL. This was achieved by combining the sample with a reagent solution consisting of 0.6 M sulfuric acid, 28 mM sodium phosphate, and 4 mM ammonium molybdate in a volume of 3 ml. The reaction mixture was subjected to incubation at a temperature of 95℃ for a duration of 90 min. within a water bath. The absorbance of all sample mixtures was quantified at a wavelength of 695 nm. The total antioxidant activity was quantified by measuring the amount of ascorbic acid equivalents.

Scavenging of H₂O₂

The capacity of alkaloids derived from the marine actinobacterial strain to eliminate H₂O₂ was assessed using the methodology outlined by Ramasamy et al. [20], with minor modifications. A solution of 40 mM H_2_O_2_ was prepared, its concentration was analyzed using spectrophotometry, and the absorbance was measured with the extraction coefficient for H_2_O_2_. Alkaloid and a standard solution of ascorbic acid at concentrations of 25, 50, 75, and 100 were introduced into a 0.6 ml solution of 40 mM H_2_O_2_. Following a 10-minute incubation period, the absorbance of H_2_O_2_ was measured at a wavelength of 230 nm. A blank solution consisting of phosphate buffer without H2O2 was used as the reference. The scavenging % of H₂O₂ was calculated using the formula given below.

Scavenging effect (%) = (A_0_ cont − A_1_ test) A_0_ cont × 100

Here A_0_ is the absorbance of control and A_1_ is the absorbance of the sample.

## Results

Visual observation

**Figure 3:**
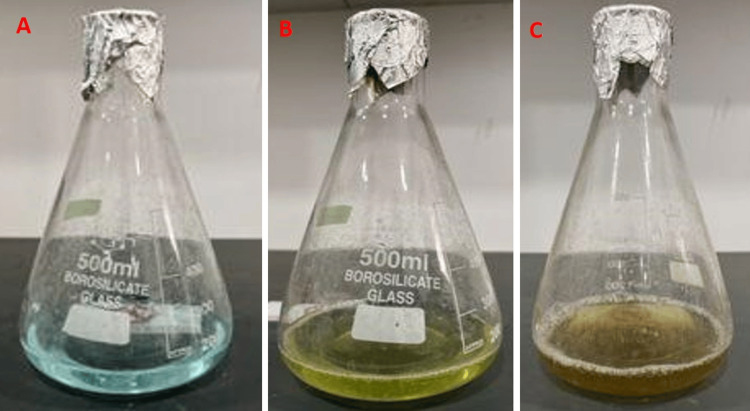
Copper solution (3A), the initial stage of copper nanoparticle synthesis (3B), and Color change after 24 hours (3C)

Figure 3 illustrates that the solution containing copper and the aqueous extract underwent a darkening process over a period of 24 hours, indicating the production of copper nanoparticles.

UV-Visible Spectroscopy of Cu Nanoparticles

**Figure 4:**
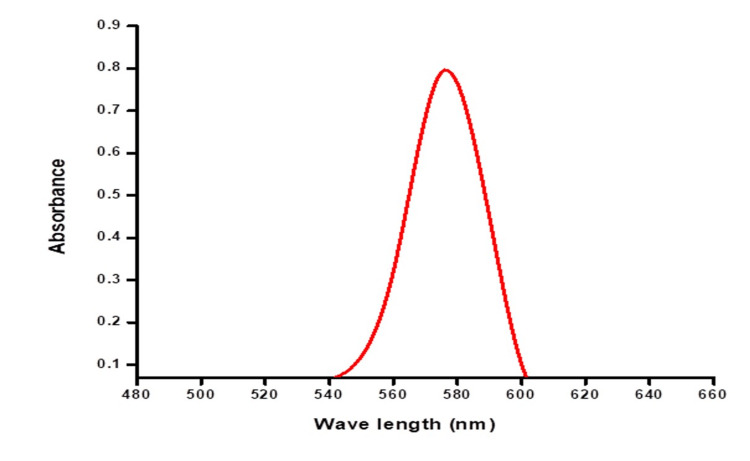
UV-visible spectroscopy of copper nanoparticles from Ulva lactuca

The graph illustrates the correlation between the absorbance level and wavelength of the Cu nanoparticles synthesized in conjunction with seaweed *Ulva lactuca*. The seaweed-derived Cu nanoparticles exhibited a peak absorbance of 1.2 at a specific wavelength of 580 nm (Fig.4).

In vitro antioxidant assays

**Figure 5:**
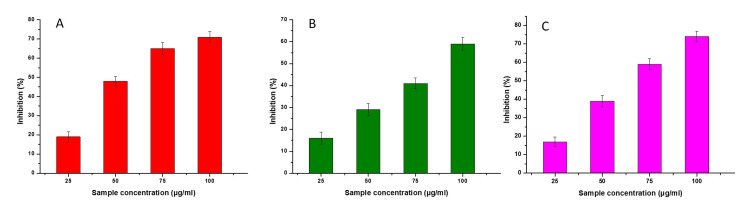
In vitro antioxidant activity of copper nanoparticles, DPPH assay (5A), total antioxidant assay (5B), and scavenging of hydrogen peroxide (5C)

Antioxidant activity was assessed using three standard methodologies, specifically DPPH assay, total antioxidant assay, and H₂O₂ assay. In this study, various concentrations of Cu nanoparticles (25, 50, 75, and 100 µg/ml) were investigated to evaluate their efficacy in scavenging free radicals. The findings derived from the DPPH assay indicated that the ability to scavenge free radicals exhibited a positive correlation with the quantity of copper nanoparticles, thus demonstrating a dose-dependent relationship. An observable increase in the level of DPPH radical inhibition was observed with an increase in the Cu nanoparticle concentration. The study findings indicate a significant antioxidant effect of the nanoparticles with regard to DPPH radicals. The total antioxidant assay and H₂O₂ assay demonstrated an increase in their capacity to eliminate free radicals that were concentration-dependent, similar to the DPPH assay. The observed capacity of Cu nanoparticles to effectively reduce the levels of H₂O₂ indicated their robust antioxidant properties (Figure 5).

## Discussion

*Ulva lactuca* has been employed as a green technique for the biomimetic manufacture of Cu nanoparticles. Based on its total antioxidant capacity and efficacy in DPPH and hydrogen peroxide scavenging assays, the synthesized copper nanoparticles exhibited notable antioxidant activity. Previous studies have demonstrated the existence of flavonoids, glycosides, phenolic compounds, saponins, steroids, and tannins in Cu nanoparticles derived from *Ulva lactuca* as reported in the literature. Various reactive oxygen species (ROS) are significantly associated with the pathogenesis of diseases, such as cancer, atherosclerosis, and diabetes. The utilization of herbal antioxidant components that produce phytochemicals can effectively protect against oxidative damage [21].

The reducing capability of seaweeds is determined by the presence of phytocomponents such as polysaccharides, phenolic compounds, proteins, enzymes, and other chelating chemicals. The marine alga *Ulva compressa* is a widely distributed species with a global presence in various heavy metal-contaminated habitats, including the northern region of Chile. *Ulva lactuca* has demonstrated the capacity to accumulate several heavy metals, rendering them a valuable bio-monitoring organism for heavy metal assessment over diverse global regions. Based on existing literature, microalgae have been firmly established as a highly promising resource for the production of biofuels and metallic nanoparticles. These nanoparticles have extensive utility in various domains, including clinical diagnostics, agriculture, and significant sectors, such as paints, electronics, coatings, and packaging. Algae-mediated metallic nanoparticle production approaches are superior to chemical synthesis methods owing to the significantly faster growth rate of algae compared to alternative sources [12].

In this study, a direct approach was employed to assess and compare the antioxidant capacities of various non-refined seed oils with those of refined seed oils. Owing to the intricate nature of oxidation reactions in both food systems and laboratory settings, the choice of methodology utilized to assess the impact of antioxidants on such reactions is crucial. The presence of antioxidants in food suggests that the digestion process may be influenced by the release of these molecules, as observed in antioxidant studies [12]. The observed maximum antioxidant activity of the Cu nanoparticles can be attributed to the presence of many bio-reductive groups of phytochemicals on their surfaces. The utilization of Cu nanoparticles in an environmentally sustainable manner can be attributed to their ability to combat free radicals and mitigate oxidative damage through their antioxidant properties. The inclusion of Cu nanoparticles into the extract resulted in a significant increase in absorbance, suggesting enhanced antioxidant efficacy [22]. The significance of accurately establishing the ideal concentration of copper nanoparticles for specific antioxidant purposes is highlighted by the dose-dependent response reported in the DPPH, total antioxidant activity, and H_2_O_2_ assays. In the present study, higher concentrations demonstrated higher levels of antioxidant activity. However, it is crucial to maintain a delicate equilibrium between effectiveness and safety. The potential for adverse consequences resulting from elevated levels of nanoparticles necessitates additional investigations to evaluate their compatibility with biological systems [22]. However, in the current investigation, no adverse effects were encountered or reported during the duration of the experiment.

The algae-mediated synthesis of metal nanoparticles has demonstrated its versatility as a material with diverse applications across several fields, notably in clinical diagnostics and biotechnology. Literature highlights the potential of algae as a viable biological tool for the production of metallic substances. The nanoparticles derived from *Ulva compressa* exhibit a transient reaction to Cu-induced stress, characterized by an upregulation in the expression of genes responsible for the synthesis of proteins related to photosynthesis, enzymes involved in carotenoid production, and genes associated with the Calvin-Benson cycle. This molecular response potentially facilitates the enhanced uptake of carbon dioxide [22].

Seaweeds are commonly utilized in industrial applications; however, their utilization for nanoparticle synthesis at the global level has not been extensively implemented. Seaweeds serve as valuable reservoirs of raw materials for a diverse range of industrial goods. There has been limited scholarly investigation on the antibacterial, antiproliferative, antifungal, and anticancer properties of silver nanoparticles derived from seaweed [22]. Numerous seaweeds possess the capacity to address a diverse array of ailments, and several seaweeds have undergone scrutiny in clinical trials, ultimately paving the way for their use in pharmaceutical production. Identification of the most efficacious seaweed species for the production of silver nanoparticles (AgNPs) and their potential therapeutic applications in combating human diseases is of significant importance. This is because of the proven effectiveness of some seaweed varieties in addressing both prevalent and multidrug-resistant illnesses in humans [23].

Oxygen permeability refers to the ability of oxygen molecules to pass through a given film under specific conditions. This property is valuable in predicting the shelf life of packaged goods. The cellulose sheets were supplemented with ulvan, resulting in reduced oxygen permeability (OP) values. The discovery of increased film compactness and reduced matrix channels can be attributed to the hydrogen bonds formed between cellulose and ulvan, as evidenced by Fourier transform infrared (FT-IR) research. These robust intermolecular interactions may have influenced the thermal stability of the films [24,25]. The maximum activity was observed when each antioxidant molecule was present at a concentration of 100 µg/ml. Based on the assessment of DPPH scavenging activity, it was observed that the crude extract, along with its aqueous component, exhibited the highest antioxidant efficacy. The results of scavenging tests indicated that the water fraction exhibited the highest efficiency. Given its typically high level of activity compared with the other fractions under examination, the water fraction was selected as a viable option for further chromatographic analysis [26].

Limitation

To make progress in this field of research, it will be crucial to overcome these restrictions by employing rigorous experimental design, conducting extensive characterization, and giving serious thought to prospective applications and safety considerations. Moreover, engaging in collaborative efforts with specialists in the fields of nanotechnology, biotechnology, and environmental research can help overcome some obstacles. Additional investigations into the antioxidant activity of the synthesized nanoparticles using in vivo animal models, specifically focusing on the liver and distinct brain regions, would contribute significant empirical support to the efficacy of this antioxidant. Apart from addressing these constraints and undertaking additional investigations in these domains pertaining to side effects or issues, it would be possible to enhance the secure and efficient advancement of copper nanoparticles for different applications.

## Conclusions

The evaluation of the overall antioxidant capacity and ability to scavenge free radicals indicates that the inclusion of Cu nanoparticles in *Ulva lactuca* has the potential to enhance their antioxidant activity. Cu nanoparticles, upon synthesis, demonstrate considerable antioxidant characteristics through efficient neutralization of free radicals and provision of defense against reactive oxygen species (ROS). This implies that they have the potential to be utilized in the treatment of disorders associated with oxidative stress and as antioxidants in a range of functional foods and pharmaceutical preparations. In conclusion, the process of synthesizing Cu nanoparticles from *Ulva lactuca* offers an ecologically conscious and enduring approach to nanoparticle fabrication. The nanoparticles that were synthesized demonstrated significant antioxidant capabilities in vitro, indicating their prospective applications in diverse domains, such as medicine and biotechnology. Additional research is required to fully realize their maximum capabilities and guarantee their safety in practical contexts.
